# Beneficial Role of Antioxidant Secondary Metabolites from Medicinal Plants in Maintaining Oral Health

**DOI:** 10.3390/antiox10071061

**Published:** 2021-06-30

**Authors:** Manoj Kumar, Suraj Prakash, Radha, Neeraj Kumari, Ashok Pundir, Sneh Punia, Vivek Saurabh, Poonam Choudhary, Sushil Changan, Sangram Dhumal, Prakash Chandra Pradhan, Omar Alajil, Sudha Singh, Neha Sharma, Tamilselvan Ilakiya, Surinder Singh, Mohamed Mekhemar

**Affiliations:** 1 Chemical and Biochemical Processing Division, ICAR—Central Institute for Research on Cotton Technology, Mumbai 400019, India; 2 School of Biological and Environmental Sciences, Shoolini University of Biotechnology and Management Sciences, Solan 173229, India; surajpandiar75@gmail.com (S.P.); neeruguleria1532001@gmail.com (N.K.); 3 School of Mechanical and Civil Engineering, Shoolini University of Biotechnology and Management Sciences, Solan 173229, India; ashok.pundir78791@gmail.com; 4 Department of Food, Nutrition, & Packaging Sciences, Clemson University, Clemson, SC 29634, USA; snehpunia69@gmail.com; 5 Division of Food Science and Postharvest Technology, ICAR—Indian Agricultural Research Institute, New Delhi 110012, India; vivek_11593@iari.res.in (V.S.); omar8alajil@gmail.com (O.A.); 6 Agricultural Structure and Environment Control, ICAR—Central Institute of Post-Harvest Engineering and Technology, Ludhiana 141004, India; poonam@icar.gov.in; 7 Division of Crop Physiology, Biochemistry and Post-Harvest Technology, ICAR—Central Potato Research Institute, Shimla 171001, India; sushil.changan@icar.gov.in; 8 Division of Horticulture, RCSM College of Agriculture, Kolhapur 416004, India; sdhumal@msu.edu; 9 Division of Agricultural Chemicals, ICAR—Indian Agricultural Research Institute, New Delhi 110012, India; prakash0844@gmail.com; 10 Department of Food Sciences, Faculty of Agriculture, Aleppo University, Aleppo 15310, Syria; 11 Department of Vegetable Science, Dr. Y. S. Parmar University of Horticulture and Forestry, Nauni 173230, India; sudhasingh230795@gmail.com; 12 Department of Biotechnology, Dr. Y. S. Parmar University of Horticulture and Forestry, Nauni 173230, India; neha.nea.sharma@gmail.com; 13 Department of Vegetable Science, Tamil Nadu Agricultural University, Coimbatore 641003, India; ilakiyatamil@gmail.com; 14 Dr. S. S. Bhatnagar University Institute of Chemical Engineering and Technology, Panjab University, Chandigarh 160014, India; ssbhinder@pu.ac.in; 15 Clinic for Conservative Dentistry and Periodontology, School of Dental Medicine, Christian-Albrecht’s University, 24105 Kiel, Germany

**Keywords:** phytochemicals, oral health, essential oils, medicinal plants

## Abstract

Plant-derived phytochemicals have been touted as viable substitutes in a variety of diseases. All over the world, dentists have turned to natural remedies for dental cure due to the negative possessions of certain antibacterial mediators used in dentistry. Antimicrobial and other drugs are currently in use, but they show some side effects. Since ancient times, antioxidant EOs have been used for different ailments and have grown in popularity over time. Several in vitro, in vivo, and clinical trials have shown the safety and effectiveness of antioxidant essential oils (EOs) in oral health obtained from medicinal plants. The current review of literature provides a summary of secondary metabolites, more specifically EOs from 20 most commonly used medicinal plants and their applications in maintaining oral health. Dental caries and periodontal diseases are the most common and preventable global infectious diseases, with diseases of the oral cavity being considered major diseases affecting a person’s health. Several clinical studies have shown a connection between oral diseases and oral microbiota. This review discusses the role of antioxidant secondary metabolites in inhibiting the growth of oral pathogens and reducing the formation of dental plaque, and as well as reducing the symptoms of oral diseases. This review article contributes a basic outline of essential oils and their healing actions.

## 1. Introduction

Oral diseases are the main worldwide health complications that affect approximately 3.5 billion people worldwide due to their chronic and progressive nature. Most oral diseases can be treated in their early stages and are largely preventable. With the increasing urbanization and changes in lifestyle, mostly in developing countries prevalence of oral diseases continues to increase. The poor access to oral health care facilities in the community, having food and beverages high in sugar, and insufficient exposure to fluoride in toothpaste or water supply will be the reasons behind the increase in oral disease. The most common oral diseases that include clinical conditions affecting mouth and teeth are periodontal (gum) diseases, dental caries (tooth decay), oral cancers, oro-dental trauma, oral manifestations of HIV, cleft lip and palate, and Noma. It was reported in earlier studies that approximately 2.3 billion people suffer from dental caries of permanent teeth [1]. In earlier studies, it was reported that approximately 20% of people suffer from oral diseases [2]. The cure is lengthy and costly, which consequently results in complications for psychological and facial growth.

Having maximum efficiency and minimum harmful effects, the natural products derived from medicinal plants play a vital role in oral health complications such as bleeding gums, mouth ulcers, dental caries, gingivitis, and halitosis. The different plant species produce different kinds of secondary metabolites. According to a study, approximately 30% of entire plant species were used for medicinal purposes depending on the type and amount of secondary metabolite they contain [3]. In developing countries, drugs of plant origin have a significant role in saving the life of many peoples. Despite the advances in synthetic drugs and modern medicine still, a large sector of world residents has a dependence on plant-origin drugs [3]. According to WHO, most of the global population has a dependency on medicinal plants as health care requirements. Drugs including amine fluorides, chlorhexidine, cetylpyridinium chloride, and triclosan were commonly used in dental care products, cause staining of teeth, and are toxic if used in excess [4]. Mouthwashes made from natural products are found to be useful in the treatment of gingivitis and plaque with effective antimicrobial activity. In odontology, herbal extracts from medicinal plants were used as antimicrobial plaque agents, antioxidants, analgesics, and antivirals to prevent histamine release because of fewer side effects and low toxicity [4]. In recent years, herbal plant extract of neem leaf, burdock root, propolis, and noni fruit were used as intra-canal medications and, having effective results, provided a novel function in global dental therapy for herbal agents [5].

At the present time, there is an upsurge in demand for essential oils extracted from various medicinal plants in the pharmacological industry due to their antioxidant, antifungal, antimicrobial and antiviral properties. EOs contain a mixture of chemical composites having less molecular weight, such as terpenoids, carbonyl compounds, alcohols, aliphatic compounds, and polyphenols [6,7,8]. In recent time approximately 3000 EOs has been reported [9]. As compared to synthetic chemicals, essential oils (natural) were harmless for the atmosphere and are more effective. The essential oils were taken out from different plant parts with leaves, fruits, flowers, bark, and root by using steam distillation, solvent extraction, and hydrodistillation. The essential oil of *Zanthoxylum armatum* is commonly called *Zanthoxylum* oil and is known to treat inflammatory pain of toothache. In the pharmaceutical industry, fruit extract is used as an ingredient in toothpaste due to its antioxidant and antimicrobial properties. *Ocimum sanctum,* the sacred plant commonly called tulsi, is used for medicinal purposes. The essential oil of *Ocimum sanctum* possesses antimicrobial, antifungal properties against oral pathogens known to cause dental problems and is used as an ingredient in mouthwash, toothpaste by pharmaceutical industries to treat toothache and pupiltis. Eugenol, one of the extensively used compounds in dentistry, is also present in the essential oil extracted from the leaves of tulsi. EO of *Salvadora persica* (miswak) is used extensively in mouthwash, toothpaste, dental varnish, dental cement due to the bioactive compounds present in it. The essential oil of miswak is reported to have antigingivitis, orthodontic chain preservation, promotion of gingival wound healing, antiplaque, anti-cariogenic, and whitening properties [10]. The essential oil of *Eucalyptus globulus,* commonly called eucalyptus oil, contains the biologically active compound eucalyptol and is used in dentistry for mouthwash and dental preparations as an endodontic solvent [11]. Thyme oil extracted from *Thymus vulgaris* reported to have antimicrobial properties and is used as an antiseptic mouth wash, toothpaste, and treatment of oral infection [12].

**Figure 1:**
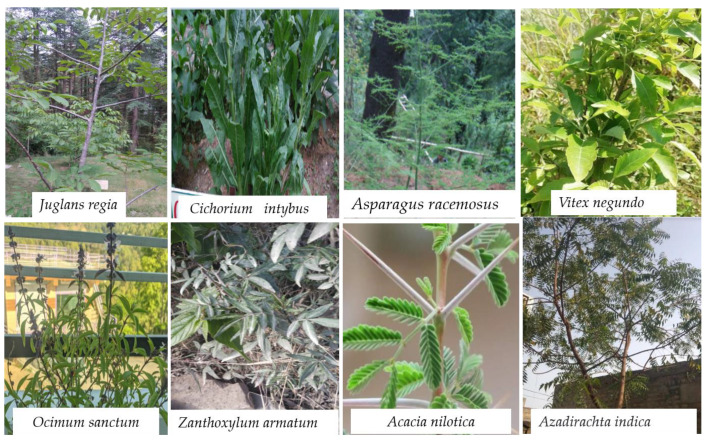
Photographs showing important medicinal plants with a beneficial role in oral health.

All over the world, oral infections persist in being a key health issue. It was found that dental caries, oral tissue lesions, and oral cancers are dangerous diseases that are the greatest chief global oral health complications. Oral fitness is very important to overall well-being. In earlier studies, it was found that a strong connection between activities of the microbiota of the oral cavity and oral illnesses. About 750 bacterial species are responsible for oral illnesses [13]. All over the world, there is a requirement for alternative preventative options, treatments, and products for oral illnesses that are safe and highly effective [14,15]. This review summarizes existing available data on the subject of medicinal usage, phytochemical composition, and pharmacological properties and evaluates the possible opportunities to use essential oils for oral infections. The EOs are less toxic, and they contain biologically active compounds having medicinal properties due to which in the last few years, there is an increase in demand, especially in pharmacological industries related to dentistry, therefore a systematic review of the phytochemical composition of EOs, and medicinal properties can help the students, researchers, and stakeholders in the development of new products to treat oral health problems such as periodontitis, dental caries (tooth decay), gingivitis. From this time, the search for other possible products continues, and naturally available chemicals extracted from medicinal plants used in traditional medicine are considered as suitable alternatives to commercially available chemicals. The products derived from different medicinal plants such as *Azadirachta indica*, *Thymus vulgaris*, *Asparagus racemosus*, *Juglans regia,* and *Ocimum sanctum* possess different types of phytochemicals and some used in pharmaceuticals [16,17,18,19,20,21,22]. The medicinal plants discussed in the current review are presented with their systematic classification in Table 1, their role in oral health is given in Table 2, and photographs of few discussed plants are shown in Figure 1. This review presents a comprehensive compilation of traditional medicines or phytochemicals extracts that inhibit the growth of oral pathogens, dental plaque and decrease the warning sign of oral illnesses. Further, the review also explores the information related to antioxidant EOs and their beneficial role in improving oral health. It was observed that very few studies have been available for an oral health cure.

## 2. Methodology

**Figure 2:**
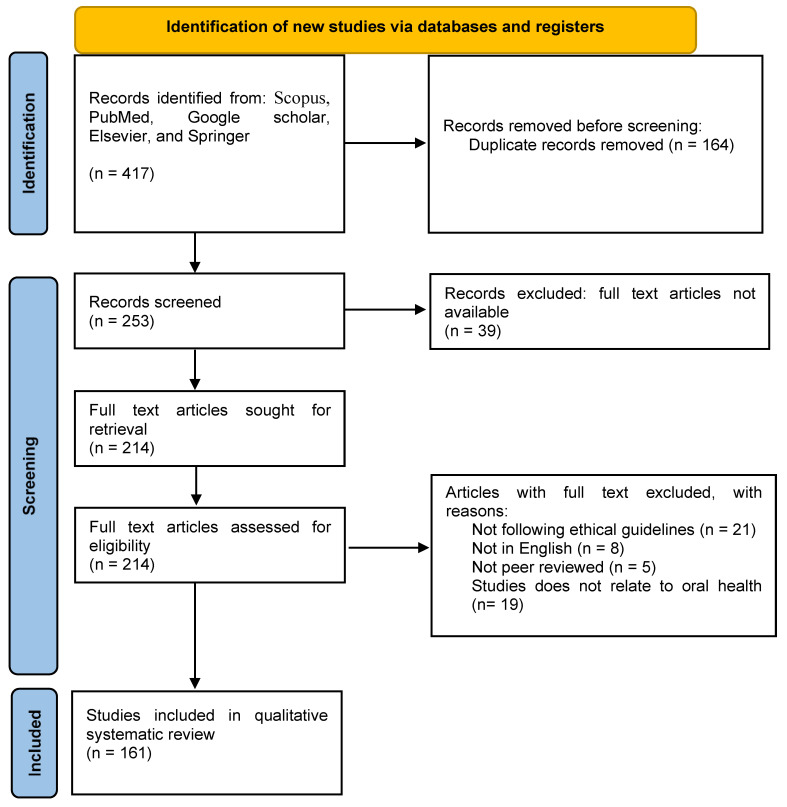
PRISMA flow diagram for the selection process of studies included in qualitative systematic review.

The current review focus on the beneficial effect of secondary metabolites derived from medicinal plants in oral health. The eligibility of studies includes the following inclusion criteria: (i) medicinal plants that were having less reviewed literature and found rarely were selected; (ii) studies published in the English language were included; (iii) original studies were selected that examined the efficacy of essential oils in oral health; and (iv) in vivo and clinical trial studies were selected on the basis of authenticity. Exclusion criteria: (i) studies not published in English; (ii) in vivo studies not followed ethical guidelines; (iii) studies not available in full text. A literature search is carried out on Scopus, PubMed, Google Scholar, Elsevier, and Springer using the following keywords in combination: oral health, essential oils, medicinal plants, phytochemicals, periodontitis, dental caries, dental plaque, gingivitis, in vitro, in vivo studies, clinical trials, microbial infections. The last search was performed on 10 May 2021. A total of 417 records were found during database searches. In the first filter, a total of 164 duplicate records were removed. Then, articles that follow eligibility criteria were selected. The following data were collected from studies:

The names of plants were followed according to the plant list [97]. The essential oil composition of plant parts, their extraction method, and solvent used. In vitro, in vivo studies, and clinical trials: effect of EOs on various infections, the concentration of EOs used, study subjects, method of application. For the selection, Preferred Reporting Items for Systematic Reviews and Meta-Analyses (PRISMA) 2020 guidelines were followed [98]. The selection process, including identification, number of records identified, eligibility criteria, and screening, is demonstrated in PRISMA flow diagram Figure 2.

## 3. Medicinal Plants and Beneficial Role in Oral Health

### 3.1. Zanthoxylum armatum DC. (Tejphal) 

*Zanthoxylum armatum* belongs to the family Rutaceae. Globally, *Zanthoxylum armatum* is found in countries such as Nepal, Malaysia, the Philippines, China, Pakistan, Japan, and Taiwan at an altitude range of 1300 to 1500 m [23]. It is distributed in India from Kashmir to Bhutan up to an altitude of 2500 m [99]. *Zanthoxylum* species are used to treat dental disorders, which include *Zanthoxylum armatum DC.*, *Zanthoxylum acanthopodium DC.*, *Zanthoxylum alatum Roxb.*, *Zanthoxylum capense (Thuanb.) Harv*., *Zanthoxylum americanum Mill*., *Zanthoxylum macrophylla Engl*., *Zanthoxylum rhetsa Roxb*., *Zanthoxylum rhoifolium Lam.*, *Zanthoxylum xanthoxyloides Waterm*, and *Zanthoxylum chalybeum* Engl. [100,101]. Different plant parts of *Zanthoxylum* species such as leaves, fruits, stems, bark, seeds, and roots are found to be enriched with various secondary metabolites viz. alkaloids, sterols, phenolics, lignins, coumarins, and terpenoids. The bark powder of *Zanthoxylum armatum,* upon mixing with honey, gives relief against gum bleeding. The extract of the toothache tree (*Zanthoxylum*) is used for the treatment of inflammatory pain (toothache) [25].

EOs of *Zanthoxylum armatum* is extracted from leaves, and analysis performed through gas chromatography. Major constituents in *Zanthoxylum armatum* such as linalool (53.05%), bergamot mint oil (12.73%), limonene di epoxide (11.39%), α-pinene (4.08%), β-myrcene (3.69%), and β-limonene (3.10%) were reported in earlier studies [23]. *Staphylococcus aureus* has been reported as a pathogen causing infections related to dental implants [102]. Antibacterial activity of EOs extracted from the leaf of *Z. armatum* is found to be effective against all tested bacterial strains, i.e., *Escherichia coli*, *Pseudomonas aeruginosa*, *Micrococcus leutus*, *Staphylococcus aureus*, *Pasteurella multocida*, *Bacillus subtilis*, and *Streptococcus viridians* [103]. The antibacterial activities of EOs of *Zanthoxylum armatum* might be due to the presence of terpenoids [24]. The hydroxy-α-sanshool alkyl amide extract of *Zanthoxylum* plants inhibits neurons that mediate sharp acute pain and inflammatory pain by blocking voltage-gated sodium channel. This is consistent with its analgesic effects in humans. Under naive and inflammatory conditions, sanshool treatment in mice produced a selective attenuation of mechanical sensitivity, with no effect on thermal sensitivity [104].

### 3.2. Ocimum sanctum L. (Tulsi)

*Ocimum sanctum* belongs to the Lamiaceae family, and distribution covers the entire Indian sub-continent. It has been found at an altitude range of 1800 m in the Himalayas. The primary centers of diversity of the genus *Ocimum* were located in Asia, Africa, and Brazil. The main countries of *Ocimum sanctum* cultivation are Haiti, Hungary, Comoro Islands, Bulgaria, Thailand, and India. The essential oil is extracted from *Ocimum basilicum* and *Ocimum sanactum* and examined for in vitro antibacterial activity. The result has shown that essential oil has effective antibacterial activity against aerobic and anaerobic organisms commonly present in the oral cavity [105].

Using hydrodistillation method, essential oil is obtained from dry leaves of *Ocimum sanctum*. Further analyzed by GC-MS (gas chromatography-mass spectroscopy), essential oil composition was found to be a mixture of alcohols (19.326%), oxides (3.252%), and hydrocarbons (26.463%). These hydrocarbons present in form of caryophyllene (22.265%), α-pinene (0.125%), α-caryophyllene (2.071%), α-bourboene (0.244%), α-cubebene (0.123%), copaene (1.637%) and camphene (0.121%). Alcohols are present in the form of methyl iso-eugenol (2.952%), eugenol (15.906%), and borneol (0.468%). Whereas in oxides, caryophyllene oxide (3.252%) was found in a significant amount [27]. Essential oils are extracted from fresh and dry parts of plants with steam distillation and hydrodistillation methods. Then analyzed with GC-MS, the main constituents found in the extract of dry leaves with hydrodistillation method were *cis*-sabinene hydrate (2.84%), linalool (6.69%), and *cis*-sabinene hydrate (2.84%), with 0.20% yield. The chief constituent from fresh leaves of *Ocimum sanctum* is spathulenol (13.92%), β-eudesmol (11.5%), and methyl chavicol (27.64%), with 0.18% yield (Milani et al., 2016). Eugenol is one of the active components found in *Ocimum sanctum* and is found to be responsible for therapeutic potential [106]. Eugenol is extensively used in dentistry for the treatment of toothache and pulpitis [107]. Due to the occurrence of eugenol, *Ocimum sanctum* acts as a cyclooxygenase (COX)-2 inhibitor. The leaves comprise 0.7% essential oil, including 20% methyl eugenol and 70% eugenol; hence, it provides relief from toothache problems [29]. The study on in vitro antifungal activity of essential oil extracted from *Ocimum sanctum* and its components eugenol and linalool against *Candida albicans* and *Candida tropicalis* known to cause oral candidiasis, shows the essential oil to be effective among *Candida* strains [108]. Antibacterial efficacy of *Ocimum sanctum* in ethanolic extract against *Streptococcus mutans* (pathogenic bacteria causing dental caries) was due to the presence of ursolic acid, carvacrol, and eugenol (1-hydroxy-2-methoxy-4-allylbenzene) [109].

### 3.3. Salvadora persica L. (Miswak)

*Salvadora persica* has its place in the Salvadoraceae family and is distributed among South and West Asia, Southern, East, and North Africa, and in the Arabic Peninsula. *Salvadora persica* is mostly found throughout the arid region in India and found in altitude range up to 1800 m. It flourishes in areas with readily accessible groundwater is readily such as riverbanks, waterholes, along drainage lines, and desert floodplains. It commonly occurs in grassy savannahs, in valleys, and thorn scrub. In Arabic countries, the stem of *Salvadora persica* is extensively used as decoctions and chewing sticks. *Salvadora persica* comprises compounds having antibacterial efficiency and plaque-inhibiting properties against cariogenic bacteria commonly present in the oral cavity [30]. The roots and small branches of *Salvadora persica* were used to prepare toothbrush and is found to be useful as maintainer of teeth. It is used worldwide to treat toothache and tooth cleaning [110]. Miswak has various traditional medicinal uses to treat disease associated with oral hygiene or dental care due to the presence of unique biologically active compounds, phytochemicals, and minerals [111]. *Salvadora persica* has been used in a probiotic spray, chewing gum, dental cement, chewing stick, toothpaste, aqueous extract, mouthwash, ethanol extract, dental varnish, and essential oil. It is found that miswak is effective as an antigingivitis, with whitening, orthodontic chain preservation, biocompatibility with oral cells, anti-cariogenic, promotion of gingival wound healing, and antiplaque properties [10].

Essential oil of *Salvadora persica* is extracted with hydrodistillation method from stem part. Further chemical composition is analyzed by GC-MS, and it shows a mixture of oxygenated monoterpenes (54%), sesquiterpene hydrocarbons (21%), and monoterpene hydrocarbons (11%), out of which the main constituents were α-caryophellene (13.4%), 9-*epi*-(*E*.)-caryophellene, 1,8-cineole (eucalyptol) (46%) and β-pinene (6.3%) [31]. A recent study on the essential oil of *Salvadora persica* showed that these oils are very effective as an antimicrobial agent in oral hygiene [112].

### 3.4. Eucalyptus globulus Labill. (Nilgiri)

*Eucalyptus globulus* is a type of shrubby plant that belongs to the family Myrtaceae. *Eucalyptus globulus* is commonly known as Nilgiri. There are around 700 species of the *Eucalyptus* genus, and it is widely used for various purposes, distributed among many countries such as Albina, Spain, Uganda, Cambodia, Nepal, and the United Kingdom [33]. In India, *Eucalyptus globulus* is commonly found in Andhra Pradesh, Bihar, Goa, Gujrat, Haryana, Punjab, Uttar Pradesh, Tamil Nadu, Kerala, and Karnataka [113]. The plant *Eucalyptus saligna* is used as a mouthwash gargle in the Cameroon region (a central African country) to treat toothache, sore throat, halitosis. The essential oil of *Eucalyptus globulus* extracted from leaves shows to have antimicrobial efficacy against *Staphylococcus aureus* (Gram-positive bacteria) and *Escherichia coli* (Gram-negative) found in the oral cavity [114,115]. The essential oil of *Eucalyptus globulus* was analyzed using GC-MS, and a total of 27 compounds were found. The chief compounds are β- pinene 18.54%, eucalyptol (1,8-cineole) 54.79%, α- pinene 11.46%, α-phellandrene 2.06%, gamma-eudesmol 1.20%, *para* cymene 1.60%, and β-eudesmol 4.68% [34]. One of the main components presents in the essential oil of *Eucalyptus globulus* is eucalyptol and used for mouthwash and dental preparations as an endodontic solvent [11].

### 3.5. Thymus vulgaris L. (Banajwain)

*Thymus vulgaris* belongs to the Lamiaceae mint family and is a flowering plant that can grow up to a height of 15–30 cm. It is distributed among the European countries such as Svizzera, France, Italy, Spain, Bulgaria, Ellas, and the Portuguese Republic [36]. In India, banajwain is distributed among the western temperate Himalayas and Nilgiris. Different parts of the *Thymus* species were used as a remedy to treat toothache by chewing on the affected tooth [37]. Thyme essential oil (1%) in ethanol was found to have antibacterial properties against pathogenic bacteria *Streptococcus mutans* and can be used in toothpaste as an ingredient [116].

The essential oil was obtained from the leaves of *Thymus vulgaris* through the steam distillation method. GC-MS analysis shows 24 bioactive compounds and each one having specific activity against different diseases or acting as a drug. The major compounds were found as thymol (3.82%), α-thymol (38.71%), camphene (0.13%), caryophyllene (0.915%), humulene (0.22%), α-terpineol (0.285%) and ρ-cymene (2.77%) [20]. In vitro studies on *Thymus vulgaris* essential oil shows antimicrobial activity against clinical isolates of pathogenic bacteria *Streptococcus mutans*, *Porphyromonas gingivalis*, *Streptococcus pyogenes,* and *Candida albicans*. Hence, have antimicrobial properties of essential oil that can be considered to use in aromatherapy for treatment and prevention of oral infections, toothpaste, and mouth rinse [12].

### 3.6. Azadirachta indica A. Juss. (Neem)

Commonly acknowledged as a holy medicinal plant, *Azadirachta indica* (neem), an evergreen tree belonging to the family Meliaceae, has been widely used in several medicinal treatments. It grows mostly in thorn forests and dry environments throughout India [117]. Neem nurtures well in the altitude range of 1500 m and is distributed among various countries having dry zones such as Afghanistan, Pakistan, India, Sri Lanka, Saudi Arabia, and tropical Africa [39,118]. Various toothpowders and toothpastes contain neem bark as a constituent due to its antibacterial properties. Studies have shown neem oil and bark to be useful in dental, gum health, or to treat dental plaque [40,119]. The study shows *Azadirachta indica* has been used to treat several dental problems by different methods. Bark and leaf extract used to cure cavities or gum disease. Various mouthwashes use neem extract used to treat tooth decay, oral infections, prevent sore and bleeding gums. In India, stems of neem trees are used by people as chewing sticks [120].

From the seeds of *Azadirachta indica,* the essential oil was extracted by using the hydrodistillation method. The essential oil chemical composition was analyzed with GC and GC-MS. The analysis showed that the chief components are oleic acid (15.7%), 5, 6-dihydro-2,4.6-triethyl-(4H)-1,3,5-dithiazine (11.7%), eudesm-7(11)-en-4-ol (2.7%) hexadecanoic acid (34.0%) and methyl oleate 3.8% [16]. The essential oil extracted from leaves of *Azadirachta indica* was extracted through steam distillation method and solvent extraction method using two solvents: ethanol and hexane. The chemical composition analyses were performed using GC-MS. The main component in steam extracted essential oil were found as diacenaphtho [1,2-j:1′,2′-1] fluoranthene (11.301%), phenol, eicosane (9.76%), (3Ar,6S,9ar)-1,2,3,4,5,6,7,9a-octahydro-8-methyl-3a, 6-metha no-3ah-cyclopentacycloocten-10-one (36.88%) and 4-((4-methoxyphenyl) methylene) amino)- (11.84%). The main compounds in ethanol extracted essential oil were found as diacenaphtho[1,2-j:1′,2′-l] fluoranthene (13.51%), butanamide, eicosane (10.259%) and *N-*(2-methoxyphenyl)-3-oxo- (16.615%). In the hexane extracted essential oil: n-hexadecanoic acid (14.688%), 9,12,15-octadecatrienoic acid, (Z,Z,Z)- (34.719%), and (3Ar,6S,9ar)-1,2,3,4,5,6,7,9a-octahydro-8-methyl-3a,6-methano-3ah-cyclopentacycloocten-10-one (10.72%). It was reported that eicosane has antifungal, antibacterial, antitumor, and cytotoxic properties [121].

### 3.7. Acorus calamus L. (Sweet flag or Vacha)

*Acorus calamus* is commonly known as vacha. It is a tall perennial wetland plant that belongs to the family Acoraceae. Traditionally, leaves and rhizome of vacha were used as medicine to cure various diseases. *Acorus calamus* is distributed among central Asia, Eastern Europe and grows well at an altitude of about 2200 m. In India, it occurs throughout different states Jammu and Kashmir, Manipur, Nagaland, Uttar Pradesh, Andhra Pradesh, Tamil Nadu, Maharashtra, Uttarakhand, Karnataka, and Himachal Pradesh [19]. *Acorus calamus* rhizome extract is used by communities of Tirumala hills to cure dental disorders [44]. The aromatic oil extracted from rhizomes of *Acorus calamus* is used traditionally for medicinal purposes [122]. It is reported that the rhizome part of *Acorus calamus* possesses numerous pharmacological activities such as CNS depressant, sedative, hypolipidemic, antimicrobial, anticonvulsant, anti-inflammatory, cryoprotective, immunosuppressive, anticancer, and antioxidant [19]. Recently an in vivo study was conducted on munident a herbal dentifrice having *Acorus calamus* as an ingredient was found to be effective in reducing gingival bleeding index score and *Streptococcus mutans* count [123]. The essential oil is extracted from the leaves of *Acorus calamus* using the steam distillation method. The chemical composition analyzed using GC and GC-MS has shown a 0.14% (v/w) yield of essential oil. The main chemical compounds of essential oil were reported as α-asarone 16.54%, γ-cadinene (3.0%), (E)-methylisoeugenol (5.06%), α-pinene 2.96%, citronellal (2.82%), followed by acoradiene (0.10%), tridecanol (0.10%), 2-(acetylmethyl)-(+)-3-carene (0.12%), 7-hexadecyne (0.13%), and rosoxide (0.14%) [42].

### 3.8. Juglans regia L. (Walnut, Akhrot)

*Juglans regia* (akhrot), belonging to the Juglandaceae family, is known to have various pharmacological activities. It is distributed throughout countries such as China and the United States. In India, it occurs in Himachal Pradesh, Uttarakhand, Jammu and Kashmir, and Arunachal Pradesh. Bark part is used for many medicinal purposes such as oral cavity hygiene, treatment of gingivitis, dental plaque, and cleaning of teeth [21]. It was found that different plant parts of *Juglans regia* possess various medicinal activities such as anthelmintic, diuretic, detergent, laxative, astringent, depurative, and exhibit antimicrobial activity. The tooth pain cure by putting a green husk piece into a hollow tooth. For dental complaints, decoction of stem bark is used [124]. The *Juglans regia* has potential use in oral hygiene products due to its antimicrobial properties due to the occurrence of terpenoids, alkaloids, steroids, phenolic compounds, and flavonoids [125]. The study on chemical composition in *Juglans regia* essential oil found 29 components with a yield of 84.25% of total essential oil. The essential oil was extracted from walnut leaves using the hydrodistillation method, and further composition was analyzed using Gas chromatography-mass spectrometry (GC-MS) and gas chromatography-flame ionization detector (GC-FID). The analysis showed main compounds in essential oil were β-pinene (2.8 to 9.5%), caryophyllene oxide (16.9 to 27.4%), germacrene (1.2 to 9.4%), and β-caryophyllene (4.0 to 22.5%). The essential oil of leaves was found to be rich in oxygenated sesquiterpenes (16.9 to 27.4%), alcohols (7.6 to 27.8), and sesquiterpene hydrocarbon (13.9 to 39.6%) [45]. In a recent study, *Juglans regia* extract containing bioactive compound juglone showed antibiofilm and growth inhibitory activity for oral pathogen, i.e., *Porphyromonas gingivalis.* In vivo study on the extract of septa and leaves showed low toxicity in mice and rats [126]. In vitro antimicrobial study shows *Juglans regia* to have antiplaque activity against pathogenic microorganisms associated with dental caries: *Streptococcus sobrinus*, *Actinomyces viscosus,* and *Streptococcus mutans.* Based on antiplaque activity *Juglans regia*, it is suggested as one of the potential products for improving oral hygiene and dental health [127].

### 3.9. Asparagus racemosus Willd (Satavari)

*Asparagus racemosus* grows up to two meters tall, favors to grow on rocky soil with an altitude range of 1300–1400 m, and belongs to the Liliaceae family. It is commonly known as satavari. It is distributed throughout Asia, Australia, and Africa at low altitudes in tropical and shade climates. In India, *Asparagus racemosus* is used for various medicinal uses. Root parts of *Asparagus racemosus* can be used as a drug to cure diseases [17]. Now, these days, oral infectious diseases such as periodontal disease and dental caries are more of the main health problems globally. The study on the chemical composition of *Asparagus racemosus* essential oil was found to have 55 compounds. The essential oil extracted from aerial parts of *Asparagus racemosus* using the solvent extraction method, and further composition was analyzed by GC-MS. The main chemical components of essential oil are myrtanol (13.72%), borneol (26.40%), 2-ethylhexanol (1.76%), pinocarveol (2.37%), perillaldehyde (8.97%), hexanal (1.34%), furfural (1.17%), decanoic acid (4.19%), undecanoic acid (2.72%), 4-(1-hydroxyethyl) benzaldehyde (1.55%), camphor (3.335), 6,10,14-trimethyl pentadecanone (1.71%), and (E)-4-hexadecen-6-yne (2.25%) [47,48]. In vitro study on the extract of *Asparagus racemosus* shows antibacterial activity against caries causing oral pathogens *Streptococcus mutans* and *Lactobacillus acidophilus* [50].

### 3.10. Juniperus communis L.

*Juniperus communis*, commonly known as Juniper and Aaraar, is an evergreen aromatic shrub that belongs to the Cupressaceae family. It is distributed throughout cold and temperate regions of the Himalayan regions, mainly in Kashmir, Bhutan, and western Tibet, at an altitude of 2743 to 4572 m above sea level. Four species of genus *Juniperus*: *Juniperus indica* Bertol., *Juniperus recurve* Buch.-Ham. ex D. Don., *Juniperus squamata* Buch.-Ham. ex D. Don., and *Juniperus communis* L., were reported from Uttarakhand [128]. The bark part of *Juniperus procera* Hochst. ex Endl. is used to cure toothache in Ethiopia [129]. A recent study reported that a correlation is found between the quantity of phosphate and calcium ions in the *Juniperus communis* toothpaste. The toothpaste of *J. communis* was linked with high phosphate concentration due to the presence of pyrophosphate in its composition. The antioxidant effect of *Juniperus communis* is reported to prevent the biological system from oxidative damage caused by a reactive form of oxygen (H_2_O_2_ and OH) [130]. The study on the species of *Juniperus communis* reported the plant to have potential use in dental practice. The plant can be used as an effective antimicrobial agent due to the availability of terpene in essential oil composition [131].

The essential oil of *Juniperus indica* and *Juniperus communis* was extracted from berries and leaves by using the hydrodistillation method. The chemical composition in essential oil is analyzed using GC-MS. In *Juniperus communis,* the essential oils of leaves and berries, a total of 48 and 59 compounds were reported with a yield of 91.24% and 87.02%, and in *Juniperus indica,* 36 and 39 compounds were reported with a yield of 91.50% and 93.77%. The main compounds reported in berries and leaves of *Juniperus* *indica* are terpinen-4-ol (16.11% and 23.61%), α-pinene (6.34% and 8.82%), sabinene (27.75 and 23.17%), and γ-terpinene (6.05% and 6.58%). In contrast, the main components of *Juniperus* *communis* leaves and berries were α-pinene (35.35% and 10.78%), limonene (23.75% and 15.06%), and terpinen-4-ol (0.93% and 8.76%) [128]. Antibacterial in vivo study on essential oil of *Juniperus communis* reported moderate to high activity against pathogen bacteria *Staphylococcus aureus* (cause of dental implant infections), *Escherichia coli*, *Hafina alvei* for concentration 5 mg/mL with a zone of inhibition 10–35 mm [102,132].

### 3.11. Melaleuca alternifolia (Maiden and Betche) Cheel

*Melaleuca alternifolia* (tea tree) is a tall shrub that normally grows 4 to 6 m in height and belongs to the Myrtaceae family. The species of the *Melaleuca* genus are distributed commonly in Australia, mostly at an altitude range of 300 m [51]. *M. alternifolia* is also reported in the Nilgiris district of Tamil Nadu, India [133]. *Melaleuca alternifolia* is used by traditional Australian medicine. For chemical compounds such as cineol, terpinen-4-ol, terpinolene, cymene, limonene, sabinene, terpinene, pinene viridiorol, and globulol, their chemical compositions include mostly terpenic compounds [52]. *Melaleuca alternifolia* is used for periodontitis [54], bad breath, relief from bleeding gums and plaque [55]. The *Melaleuca alternifolia* essential oil is extracted from leaves and terminal branches by using the steam distillation method. The main chemical component present in the essential oil extract of leaves were terpinolene, terpinen-4-ol, cineol, limonene, cymene, terpinene, pinene, sabinene, viridiflorol, and viridiflorol. Its chemical composition includes mostly terpenic compounds [52]. Recently, in vivo trials conducted on melaleuca gel were reported to have an inhibitory effect on bacterial growth, causing dental caries, periodontitis, dental plaque, and gingivitis. A study found that during experimental oral candidiasis, mice are protected by terpin-4-ol, which is one of the main chemical constituents present in *Melaleuca alternifolia* essential oil [134]. *Melaleuca alternifolia* Cheel is used in Australian traditional medicine. The essential oil extracted from leaves exhibits chemical compounds such as terpinene, terpinolene, terpinen-4-ol, cymene, cineol, limonene, pinene, sabinene, viridiflorol, and globulol. Its chemical composition exhibits mostly terpenic composites [135,136].

### 3.12. Acacia nilotica (L.) Delile

*Acacia nilotica* is an evergreen tree commonly known as Babul that belongs to the Leguminosae family. Gamble (1918), in his “Flora of Madras Presidency” book, has documented more than 40 species of *Acacia* genus from India. It is distributed among various countries such as India, Saudi Arabia, Oman, Iran, Israel, Nepal, Pakistan, Angola, Egypt, Mali, Ethiopia, Ghana, Kenya, Libya, Malawi, Botswana, Mozambique, Kenya Niger, Senegal, Somalia, Nigeria, South Africa, Tanzania, Uganda, Zimbabwe, and Sudan [56]. The essential oil is extracted using the hydrodistillation method, and GC-FID and GC/MS were used for the analysis of the constituents of essential oil. The amount of essential oil obtained from the bark of *Acacia nilotica* was 0.08% *v/w*. About 36 chemical compounds were reported in the essential oil of *A. nilotica*, out of which limonene (15.3%) and menthol (34.9%) are among the two important compounds. Monoterpenoid compounds (69.6%) are predominant in the oil as compared to sesquiterpenes (19.4%). The oil consists of the monoterpenoids limonene (15.3%) and menthol (34.9%) in higher amounts, followed by carvacrol (4.1%) and α-curcumene (6.9%) were present in small quantities [57]. Different alkaloids are present in the extract of *Acacia nilotica*, such as dimethyltryptamine, tryptamines and *N*-methyltryptamine. Using the agar diffusion technique, the antibacterial activity of stem and bark extract of *Acacia nilotica* was studied against oral pathogens: *Staphylococcus aureus*, *Streptococcus viridans*, *Escherichia coli*, *Shigella sonnei*, and *Bacillus subtilis,* and the result shows minimum inhibitory concentration (MIC) of bark and stem extract ranged in between 30 and 50 mg/mL [137]. The stem and bark part of *Acacia nilotica* can be used in toothpaste and tooth cleaner. The paste of stem and bark of *Acacia nilotica* is used to make the gum strong or to cure gum bleeding. The extract prepared from the stem and bark of *Acacia nilotica* is used in gargling to cure throat-related problems and is also helpful in relieving toothache. Its branches were also used for cleaning teeth [58]. In the traditional system of medicine, the combination of the bark of mango and the bark of *Acacia nilotica* taken in equal quantity (~6 g) boiled in water (approximately 750 mL) for half an hour and filtered is used for gargling and is helpful to cure mouth ulcers. The extract of *Acacia nilotica* provides relief from toothache, and branches are used for cleaning teeth [58]. In addition, the decoction prepared from the leaves and bark of *Acacia nilotica* in combination with the bark of *Terminalia chebula* (hardh) is used to treat mouth ulcers [56] and to cure sore throat [60].

### 3.13. Quercus infectoria G. Oilvier

*Quercus infectoria*, a small tree also known by the name of the Aleppo oak, is a species of oak that belongs to the family Fagaceae. It is native to Greece, Iran, Turkey, Persia, Cyprus, Syria, Nepal, and Asia Minor. It is distributed among some parts of India (Garhwal Himalayas). It is also known as “baloot” in Iran and is a frequently used medicinal plant. Manjakani is another name used in Malaysia for *Quercus infectoria* [61]. The main chemical constituents present in galls of *Quercus infectoria* were 50–70% tannins [138], sugar [62], gallic acid (2–4%), and ellagic acid [61]. Numerous tannins have been reported to have antibacterial efficacy against different strains of bacteria [139,140,141]. The essential oil from the leaves of *Quercus infectoria* was extracted by steam distillation method by using a Clevenger apparatus. The results show a 0.2% yield of essential oil extracted from leaves of *Quercus infectoria* [61]. *Quercus infectoria* bears galls, which are used by the traditional system of medicine since ancient times in Asia [62]. The galls occur on the branches of this tree due to the deposition of eggs by *Cynips gallae tinctoriae* (gall wasp) [142]. In India, galls of *Quercus infectoria* is used by the traditional system of medicine as a constituent of toothpaste or toothpowder for the treatment of oral cavity and gum infections. From recent studies in past years, it has been reported that galls possess antiviral, antifungal, antibacterial properties and are used for the treatment of gingivitis and toothache [62]. In the traditional system of medicine, *Quercus infectoria* is used as a tonic for teeth and gums, and for the treatment of dental cavities due to its antimicrobial property [143]. The acetone and methanol extract of the gall of *Quercus infectoria* in agar-well diffusion assay exhibited activity against oral pathogens such as *Fusobacterium nucleatum* ATCC 25586, *Streptococcus mutans* ATCC 25175, *Streptococcus salivarius* ATCC 13419, and *Porphyromonas gingivalis* ATCC 33277. The MIC ranged between 0.16 and 0.63 mg/mL, and the most susceptible bacteria is *S. salivarius*, which suggested that oak extract might be used in contradiction of periodontitis etiological agents and dental caries [62].

### 3.14. Artemisia dracunculus L.

*Artemisia dracunculus* belongs to the family Compositae and is a perennial herb, also known by the name of French tarragon and estragon. The plant grows in wild habitat throughout central Europe and Asia and is widely cultivated due to the popularity of tarragon-vinegar-based dressings and sauces. *Artemisia* possesses bioactivity due to the occurrence of numerous active ingredients (essential oil components) and secondary metabolites and has widespread pharmacological activities [144]. The extracts obtained from *A. dracunculus* have antiseptic, stimulant, antibacterial, and antifungal activities [145]. The essential oil is produced in glandular hairs and oil canals of *Artemisia dracunculus,* having a gentle, spicy scent. The essential oil mainly consists β-pineneocimene, methyl chavicol (about 65%), camphene, sabinene, limonene, and menthol. The essential oil composition of *Artemisia dracunculus* has been intensively examined, and the following compounds were identified as major chemical compounds (approximately ≥10%): estragol (methyl chavicol), α-terpinolene, (E)-anethole, capillene, methyl eugenol, (Z)-artemidin, elemicin5-phenyl,1,3-pentadiyne, (E)-α-ocimene, (Z)-β-ocimene limonene, pulegone, (E)-β-ocimene, α-phellandrene, α-pinene, isoelemicin, β-phelland-rene, acenaphthene, and hinokitiol [63]. From the aerial parts of *Artemisia dracunculus*, essential oil is obtained using the hydrodistillation method, and the GC-MS method is used for the identification of chemical constituents present in essential oil [61]. In Tajikistan, aerial parts from the *A. dracunculus* were collected, and about 45 compounds representing about 99.8% of the total fraction of oil were identified. In *Artemisia dracunculus*, limonene (7.8%), sabinene (29.1%), (Z)-artemidinn (4.9%), estragole (24.6%), (E)-β-ocimene (4.0%), and myrcene (4.8%) are present in higher amounts in the aerial parts [63]. *Artemisia dracunculus* has been used as a folk remedy since ancient times. Ibn al-Baitar, Avicenna, Al-Beruni, Gelenus, and others have reported the medicinal properties of *Artemisia dracunculus* in their research studies. Avicenna reported that tarragon grass (fresh) is useful for bad breath and bleeding gums [63].

### 3.15. Streblus asper Lour

*Streblus asper* belongs to the Moraceae family, is a small tree, and is commonly known by numerous names such as barinka, koi, berrikka, rudi, serut, Siamese rough bush, sheora, and most commonly, it is known by the name of “toothbrush tree”. It is widely distributed among several Asian countries, such as Sri Lanka, Southern China, India, the Philippines, Malaysia, and Thailand [65]. Analysis of the compounds present in the aerial parts of *Streblus asper* is performed by and HPTLC (high-performance thin-layer chromatography), TLC (thin-layer chromatography) method, and the following chemical compounds were identified in the stem part: α-amyrin acetate, β-sitosterol, strebloside, lupeol acetate, diol, sioraside, α-amyrin, mansonin, (7’S, 8’S)-threo-streblusol B, streblusquinone, (8R, 8’R)-streblusol D, (7’S, 8’S)-trans-streblusol A, streblusol E, and 8’R-streblusol C. From the aerial bark: n-triacontane, β-sitosterol, stigmasterol, tetraiacontan-3-one, oleanolic acid, and botulin, and from heartwood: flavonoids and lignans were reported [65]. Different plant parts of *Streblus asper* were used for the treatment of different ailments in folk medicines. The extract from *Streblus asper* stem bark is used to provide relief from toothache and has anti-gingivitic properties. The branch part of *Streblus asper* is used as a toothbrush for gum strengthening. The milky juice obtained from *Streblus asper* bark shows an antiseptic property that is useful as anti-infectious gargles [65]. A study reported antibacterial activity in leaf extract of *Streblus asper* is helpful in controlling dental caries [146]. In vivo study was carried out on 30 human cases, and the results revealed that one minute rinse with about 20 mL of *Streblus asper* extract (SAE) of concentration 80 mg/mL can considerably decrease the number of *Streptococcus mutans* colonies compared with water (distilled) and there is no change in the buffer capacity and salivary pH [147]. The extract of *Streblus asper* is also effective against the growth of *Porphyromonas gingivalis* and *Aggregatibacter actinomycetemcomitans* colonies [146]. *Streblus asper* leaf extract has a positive effect during subgingival irrigation in chronic periodontitis [148].

### 3.16. Cichorium intybus L.

*Cichoriumintybus* L. belongs to the Compositae family and is a perennial, woody, herbaceous plant commonly known as chichory [149]. Distributed among different parts of the world, likewise as in Afghanistan, India, Bulgaria, Italy, Morocco, Iran, Serbia, Jordan, and Poland [68]. Essential oil is extracted from aerial parts of *Cichorium intybus*, and the hydrodistillation and liquid-liquid extraction methods (a two-step process) are used. For extraction of volatile oil, pentane as solvent is used. The concentrated extract is yellow in color and has a strong smell. For the extraction and identification of constituents present in the essential oil combination of GC-FID and GC-MS methods are used, and 20 chemical compounds were identified from the aerial parts of *Cichorium intybus.* The major components present in oil are: cinnamic aldehyde (12.4%), carvacrol (50.1%), camphor (4.4%), carvone (4.1%), linalool (3.9%), terpineol (2.1%), and thymol (13.3%) [69]. The major compound present in the methanolic extract of *Cichorium intybus* is chicoric acid. Terpenoids constitute the minor portion of *Cichorium intybus,* while aliphatic composites and their byproducts constitute the major fraction. Saccharides, flavonoids, essential oils, methoxy-coumarin cichorine, and anthocyanins present in the flower of *Cichorium intybus* provide blue color to the petals [69,150]. The compounds present in the essential oil of *Cichorium intybus* (adapted from [150]) are octane, *n*-nonanal, 2-Pentyl furan, octen-3-ol, allo-aromadendrene, acetaldehyde, (2E,4E)-heptadienal, (2E,4E)-decadienal, camphor, (2E)-nonen-1-al, (2E,6Z)-nonadienal, *n*-decanal, *n*-decanol, (2E,4E)-nonadienal, (2E,4Z)-decadienal, (E)-caryophyllene, *n*-tridecane, (E)-*β*-farnesene, *β*-elemene, benzene, (2E)-tridecanol, *n*-octadecanol, (5E,9E)-farnesyl acetone, (E)-2-hexylcinnamaldehyde, *n*-nonadecane, *n*-eicosane, *n*-heneicosane, and octadecane [71]. In Cuetzalan, the latex obtained from *C. inhybtus* is used to break up molars (with cavities) by setting a drop of latex directly on the tooth. Low molecular mass (LMM) extract of *Cichorium intybus* var. *Silvestre* (red chicory) is reported to prevent virulence-linked properties of bacterial species (oral pathogens) such as *Prevotella intermedia*, actinomyces, and *Streptococcus mutans*, which are responsible for biofilm formation (plaque) and pathogens causing gingivitis and tooth decay. Succinic and quinic acid are most effective against oral pathogens that are mainly responsible for biofilm formation (by interfering with their development or promotion of commotion). From recent in vivo studies, it is reported that one or few other compounds may control plaque formation, which is responsible for the development of gingivitis and dental caries [71].

### 3.17. Vitex negundo Linn. (Nirgundi)

*Vitex negundo* L. belongs to the Verbenaceae family. In India, it is locally known by the name “Nirgundi” and is a valuable medicinal plant. *Vitex negundo* is a woody, aromatic, erect, deciduous shrub, growing up to a height of 2–5 m. It flourishes best in moist places and along water routes in wilds at an altitude of 1500 m above sea level. *V. nigundo is* distributed in India, Madagascar, Thailand, Sri Lanka, Afghanistan, Pakistan, Eastern Africa, and Malaysia [72]. GC-MS method is employed to examine the chemical compounds of essential oil obtained from the dried fruits, flowers, and leaves of *Vitex negundo.* The main components present in the essential oil were sesquiterpenes (44.41%) comprised of caryophyllene oxide (11.33%), eremophilene (12.76%), caryophyllene (18.27%), *β*-bisabolene (0.94%), *α*-bergamotene (0.53%) and humulene (0.58%). The monoterpenes (19.25%) in the oil include 1(*R*)-*α*-pinene (0.21%) and sabinene (19.04%). There are four types of esters present in the oil, named *β*-terpinyl acetate (8.99%), nerol acetate (1.18%), linalyl formate (3.72%), and 0.88% of geranyl acetate. (−)-terpinen-4-ol (2.82%), menthol (1.44%), and linalool (4.27%) are three main alcohols found in the essential oil. *o*-cymene (5.90%) is the aromatic compound, and menthone (4.96%) is the ketone found in the essential oil of *Vitex negundo*. A total of 0.40% of eucalyptol is also present in the oil [151]. The various chemical compounds present in essential oil obtained from dried fruits, leaves, and flowers are epoxide, α-cedrene, δ-guaiene, ethyl-hexadecenoate, α-selinene, β-caryophyllene (16.59%), caryophyllene epoxide, β-selinene, germacrene, hexadecenoic acid, (E)-nerolidol, guaia-3,7-dienecaryophyllene epoxide, p-cymene, valencene, viridiflorol (19.55%), 1-oceten-3-ol (1.59%), germacren-4-ol, sabinene (12.07%), γ-terpinene (2.21%), caryophyllene oxide (1.75%), 4-terpineol (9.65%), 1-oceten-3-ol (1.59%), and globulol (1.05%) [74]. The extract obtained from the stem bark and leaves of *Vitex negundo* is very useful against toothache, throat pain, mouth ulcers [152]. The decoction of *Vitex negundo* leaves is used for gargling and for the treatment of sores [76].

### 3.18. Rosmarinus officinalis L.

*Rosmarinus officinalis L.* belongs to the family *Lamiaceae*, is commonly known as rosemary, and is a medicinal plant. The plant is indigenous to Asia and South Europe, also cultivated in some parts of India and the Mediterranean basin [77]. *Rosmarinus officinalis* is also known as rusmari (Sanskrit), rosemarijin (dutch), gulmehendi (Hindi). The essential oil extracted from the leaves of *Rosmarinus officinalis* and the identification of the constituents present in the oil is performed by GC technique and HPLC (high-performance liquid chromatography), that has revealed high contents of volatile oil [78]. The principal constituents present in rosemary oil (from leaves) are: borneol (1.5–5.0%), camphor (5–31%), limonene (1.5–5.0%), camphene (2.5–12.0%), pinene (9–26%), 1,8-cineol (15–55%), pinene (2.0–9.0%), myrcene (0.9–4.5%), verbenone (2.2–11.1%), and caryophyllene (1.8–5.1%) [79]. The chief constituent present in essential oil camphor has an antibiofilm property, which helps to prevent plaque formation [79]. Recently, in vivo study reported a reduction in biofilm by rosemary essential oil and recommended its use in new anti-caries treatment protocols [80].

### 3.19. Embelia ribes Burm. f.

*Embelia ribes* Burm.f. belongs to the Primulaceae family and is commonly known as vaibidang. False black pepper is an endangered climbing shrub that generally occurs in the evergreen to semi-evergreen forests of China, India, Sri Lanka, and Malaysia [81]. In India, *Embelia ribes* grows at a 1500 m altitude range [153]. Phyto constituents present in *Embelia ribes* are embelin, embolic acid, and rapanone [81]. The berries of *Embelia ribes* comprise benzoquinone derivatives such vilangin and embelin (2,5-dihydroxy-3-undecyl-2,5-cyclohexadiene-1,4-benzoquinone) [83]. The antimicrobial efficacy of the *Embelia ribes* is due to the occurrence of secondary metabolites such as flavonoids, lectins, polyphenols, and alkaloids [153]. Embelin shows antibacterial properties against *Streptococcus mutans* and *Streptococcus sanguis* (Gram-positive bacteria) present in the mouth, responsible for biofilm formation. From *Embelia ribes*, the extraction of embelin was conducted according to principles of Indian Herbal Pharmacopoeia (2002). From *Embelia ribes* plant, 100 g of powdered berries were extracted in Soxhlet extraction for about 6 h using n-hexane as a solvent then the extract is evaporated on a rotator and is again crystallized using chloroform and ethanol, and the characterization of the extract is performed by using differential scanning calorimeter (DSC), X-ray diffraction, ultraviolet-visible spectroscopy (UV-visible), Fourier-transform infrared spectroscopy (FTIR), nuclear magnetic resonance spectroscopy (NMR), and thermo gravimetric analysis (TGA) analysis method [82]. The fruits of *Embelia ribes* were used in the ayurvedic system of medicine for medicinal purposes. In India, due to its antibacterial properties, it is traditionally employed [81]. Paste prepared from *Embelia ribes* is used as a mouthwash and is also effective against dental cavities [85].

### 3.20. Spilanthes Species

Genus *Spilanthes* belongs to the Compositae family and is distributed among the tropical regions of the world. In India, *Spilanthes* genus is characterized by the presence of six species: *Spilanthes uliginosa* Sw, *Spilanthes calva* DC, *Spilanthes radicans* Jacq., *Spilanthes paniculata* DC., *Spilanthes oleracea,* and *Spilanthes ciliate* Kunth. In the tropical and subtropical areas around the world, these plants (*Spilanthes* spp.) were used frequently in traditional medicine. The main use of *Spilanthes* spp. in the field of medicine is to treat toothache in which the leaves and fresh flowers were chewed or to relieve pain it is placed onto tooth cavities. In India, the juice of the *Spilanthes acmella* flower is effective in curing oral ulcers [86]. The main phytochemicals present in the *Spilanthes* genus are unsaturated and saturated alkyl ketones, acetylenes, hydrocarbons, terpenoids, alkamides, alkaloids, lactones, coumarins, and flavonoids. These constituents are responsible for the pharmacological activity of *Spilanthes* species [86]. Essential oils of only a few species have been explored. GC and GC-MS methods were used for the analysis of essential oils from *Spilanthes* species. The chemical composition of the essential oil is variable, indicating the presence of many chemotypes in it [86]. Supercritical (CO_2_) and SDS (simultaneous distillation extraction) from flowers, leaves, and stems of *Spilanthes americana* results in the separation of volatile compounds, which include cadinene (anisomeric hydrocarbon), sesquiterpenes (α-caryophyllene, α-and *β*-bisabolene, *β*-caryophyllene), *N*-(isobutyl)-6Z,8E-decadienamide, *N*-(2-methylbutyl)-2E,6Z,8E-de catrienamide, and *N*-(2-phenylethyl)-2E,6Z,8E-decatrienamide), and various oxygenated compounds were isolated by SDE method. Supercritical fluid extraction (SFE) extracts from the stems of *Spilanthes americana* are found rich (>40%) in sesquiterpenes, while flowers and leaves are rich in nitrogenated (43% and 27%) and oxygenated (36% and 23%) compounds. About seven chemical components from the essential oil were identified, include caryophyllene oxide, myrcene, sesquiterpene, limonene, and caryophyllene [86]. The flower part of *Spilanthes uliginosa* is used in the treatment of gum infection and sore throat [91]. The flower and leaf part of *Spilanthes acmella L.* is used for toothache and throat complaints [89]. The aerial parts of *Spilanthes filicaulis Jacq.* were useful for the treatment of tooth decay [90]. Leaves of *Spilanthes calva* is useful for gingivitis, throat complaints parts, and for toothache, teeth were brushed with flowers [92]. The decoction of leaves and flowers from *Spilanthes oleracea* is used for toothache and throat complaints [88]. The whole plant of *Spilanthes filicaulis* is effective in curing toothache. Parts used [154]. The flowers of *Spilanthes paniculate* were used for the treatment of toothache, tooth infections by chewing flowers followed by rinsing with water [155].

### 3.21. Nigella sativa L.

*Nigella sativa,* commonly known as black seed or kalonji, is well known for its health benefits [95]. Distributed among the Middle East, southern European continent, and North Africa, and in India, it is cultivated in the areas of Bengal, Bihar, Gangetic plains, Himachal Pradesh, Assam, Maharashtra, and Punjab [93]. The essential oil of *N. sativa* seed is extracted using solvent extraction method and SFE (supercritical fluid extraction), and the chemical composition of essential oil is analyzed using GC-MS. Main compound reported were carvacrol (5.8–11.6%), longifolene (1.0–8.0%), ρ-cymene (7.1–15.5%), t-anethole (0.25–2.3%), 4-terpineol (2–6.6%) and thymoquinone (27.8–57.0%) [94]. Thymoquinone (C_10_H_12_O_2_) is recognized as the most important bioactive compound found in *N. sativa* oil with medicinal properties such as antioxidant, anti-inflammatory, antimicrobial, analgesic, anticarcinogenic and antihypertensive [96]. A recent study reported that thymoquinone could play an important role in the treatment and prevention of periodontal diseases. In a clinical trial (RCTs), 0.2% thymoquinone gel shows a significant decrease in GI (gingival index), PPD (probing pocket depth), and PI (plaque index) levels and an increase in GCF (gingival crevicular fluid)–ALP (alkaline phosphatase) levels. They also show sensitivity against *Prevotella intermedia*, *Porphyromonas gingivalis,* and *Aggregatibacter actinomycetemcomitans* [95].

Role of various medicinal plants discussed in the current review against various oral pathologies is presented in Table 2. 

## 4. Antioxidant Extracts from Medicinal Plants in Oral Health: A Clinical Trial Perspective

The antigingivitic and antiplaque effect of fluoridated dentifrice and 4% *Ocimum sanctum* extract was studied in a triple blinded randomized clinical trial (RCT) among 14–15-year-old school children, and reduction in dental plaque (*p* = 0.01) and gingivitis (*p* = 0.001) was observed maximum in 4% tulsi extracts in comparison with the fluoridated dentifrice group [156]. In triple blinded RCT, the effect of phenolic mouth wash and *Salvadora persica* oral rinse was compared among girls 18–22-year-old for six months and were found to be equally effective as no statistically significant difference was observed in all the examination phases between the mean gingival and plaque scores of two groups [157]. The effect of *Eucalyptus globulus* extract added as an ingredient in herbal product (tooth and gums tonic) was compared with chlorhexidine M gel in double-blind RCT and showed a decrease in mean gingival and plaque value at different intervals. It was observed to be equally effective in comparison to chlorohexidine with no statistically significant difference (*p* = 0.001) [158].

In double-blinded RCT, the efficacy of 2.5% NaOCl is compared to neem as root canal irrigants on amount of endotoxin and intensity of pain after root canal treatment in mandibular molars with necrotic pulps. It is observed that the neem group has lower mean pain scores compared to the 2.5% NaOCl group and shows no significant difference except 24 h following instrumentation (*p* = 0.012). Endotoxin levels were reduced by 18% in neem group and 8% in NaOCl group in comparison to pre-instrumentation samples (*p* < 0.001) [159]. In a recent study (RCT), the efficacy of *Juglans regia* on developing dental plaque was examined among 16–30 years age group, and the result shows 2% ether extract (bark) with maximum plaque inhibition of 32.12% as compared to other preparations 3% ether extract 31.56%, petroleum ether extract 2–17.62% and 3–19.45%, and aqueous solution 2–30.32% and propylene glycol as a solvent in preparations shows 7.88% of antiplaque activity [46].

Recently RCT is conducted on 30 patients to evaluate the efficacy of locally delivered 5% tea tree oil (TTO) gel adjunctive to scaling root planning (SRP) as an intrapacket application for stage 2 periodontitis treatment, and significant difference and improvement was observed in biochemical and clinical parameters at *p* < 0.001 in both groups. The test group treated with 5% TTO gel and SRP is found to be more effective in treating stage 2 periodontitis in comparison with the control group treated with SRP only [160]. Table 3 presents examples of various clinical trials showing positive effect of medicinal plant extracts in maintaining oral health.

## 5. Conclusions

According to the evidence presented in this review, EOs have the potential to be used as preventive or therapeutic agents for a variety of oral diseases. Despite the fact that many other potential uses of EOs have been identified and many reports of therapeutic efficacy have been adequately validated by either in vitro testing or in vivo clinical trials, more research is needed to determine the safety and efficacy of these EOs before they are used in clinical practice. They can be very useful in dental therapy and contribute to improving the quality of dental treatments if used properly. Clinical studies that validate the therapeutic potential of EOs in vivo and discuss concerns including adverse effects, toxicity, and their interaction with other drug molecules, in particular, would be extremely beneficial. Based on the available data, it can be concluded that EOs have the potential to be developed as preventative or therapeutic agents for a variety of oral diseases, but further clinical trials are needed to confirm their safety and efficacy. There is strong evidence that plant extracts, essential oils, and extracted plant chemicals have the ability to evolve into treatments that can be used as curative agents for oral diseases, as shown by various examples included in this review. While the number of clinical trials for such drugs is promising, more research on their effectiveness would be needed to determine their therapeutic effects, either alone or in conjunction with traditional therapies. The review addresses the research issues of standardization of extracts or purified compounds, and quality control would be of great significance to obtain better dental care with the support of accessible natural wealth. This review gives an outline of essential oils, their therapeutic belongings, and their effects.

## Figures and Tables

**Table 1:** Systematic classification of the medicinal plants discussed in the current review.

Sr. No.	Common Name	Kingdom	Phylum	Class	Order	Family	Genus	Species	Binomial Classification
1.	Tejphal	Plantae	Magnoliophyta	Magnoliopsida	Sapindales	Rutaceae	Zanthoxylum	*Zanthoxylum armatum*	*Zanthoxylum armatum* DC.
2.	Tulsi	Plantae	Magnoliophyta	Magnoliopsida	Lamiales	Lamiaceae	Ocimum	*Ocimum sanctum*	*Ocimum sanctum* Linn.
3.	Miswak	Plantae	Magnoliophyta	Magnoliopsida	Brassicales	Salvadoraceae	Salvadora	*Salvadora persica*	*Salvadora persica* L.
4.	Nilgiri	Plantae	Magnoliophyta	Magnoliopsida	Myrtales	Myrtaceae	Eucalyptus	*Eucalyptus globulus*	*Eucalyptus globulus* Labill.
5.	Banajwain	Plantae	Magnoliophyta	Magnoliopsida	Lamiales	Lamiaceae	Thymus	*Thymus vulgaris*	*Thymus vulgaris* L.
6.	Neem	Plantae	Magnoliophyta	Magnoliopsida	Sapindales	Meliaceae	Azadirachta	*Azadirachta indica*	*Azadirachta indica* A. Juss.
7.	Vacha	Plantae	Magnoliophyta	Liliopsida	Arales	Acoraceae	Acorus	*Acorus calamus*	*Acorus calamus* L.
8.	Akhrot	Plantae	Magnoliophyta	Magnoliopsida	Juglandales	Juglandaceae	Juglans	*Juglans regia*	*Juglans regia* L.
9.	Satavari	Plantae	Magnoliophyta	Liliopsida	Asparagales	Asparagaceae	Asparagus	*Asparagus racemosus*	*Asparagus racemosus* Willd.
10.	Aaraar	Plantae	Coniferophyta	Pinopsida	Pinales	Cupressaceae	Juniperus	*Juniperus communis*	*Juniperus communis L.*
11.	Tea tree	Plantae	Magnoliophyta	Magnoliopsida	Myrtales	Myrtaceae	Melaleuca	*Melaleuca alternifolia*	*Melaleuca alternifolia* (Maiden and Betche) Cheel
12.	Babul	Plantae	Magnoliophyta	Magnoliopsida	Fabales	Fabaceae	Acacia	*Acacia nilotica*	*Acacia nilotica* (L.) Delile
13.	Baloot	Plantae	Magnoliophyta	Magnoliopsida	Fagales	Fagaceae	Quercus	*Quercus infectoria*	*Quercus infectoria* G. Olivier
14.	Estragon	Plantae	Magnoliophyta	Magnoliopsida	Asterales	Asteraceae	Artemisia	*Artemisia dracunculus*	*Artemisia dracunculus* L.
15.	Khoi	Plantae	Magnoliophyta	Magnoliopsida	Urticales	Moraceae	Streblus	*Streblus asper*	*Streblus asper* Lour.
16.	Chicory	Plantae	Magnoliophyta	Magnoliopsida	Asterales	Asteraceae	Cichorium	*Cichorium intybus*	*Cichorium intybus* L.
17.	Nirgundi	Plantae	Magnoliophyta	Magnoliopsida	Lamiales	Verbenaceae	Vitex	*Vitex negundo*	*Vitex negundo* L.
18.	Rosemary	Plantae	Magnoliophyta	Magnoliopsida	Lamiales	Lamiaceae	Rosmarinus	*Rosmarinus officinalis*	*Rosmarinus officinalis* L.
19.	Vaibidang	Plantae	Magnoliophyta	Magnoliopsida	Ericales	Primulaceae	Embelia	*Embelia ribes* Burm.f.	*Embelia ribes* Burm.f.
20.	Akalkara	Plantae	Magnoliophyta	Magnoliopsida	Asterales	Asteraceae	Spilanthes	*Spilanthes acmella*	*Spilanthes acmella* (L.) L.
21.	Kalonji	Plantae	Magnoliophyta	Magnoliopsida	Ranunculales	Ranunculaceae	Nigella	*Nigella Sativa*	*Nigella sativa* L.

**Table 2:** Medicinal plants useful in oral health.

Botanical Name (Common Name)	Location	Extraction Method or Type of Solvent	Essential Oil Components	Study Type (In Vitro/In Vivo/Clinical Trial) and Dose of the Extract	Role in Oral Health
*Zanthoxylum armatum* DC. (Tejphal, Tumbru)	India: Kashmir to Bhutan, China, Taiwan, Malaysia, Japan [23]	Hydrodistillation method, Analyzed-GC-MS	Linalool (53.05%), Limonene (11.39%), Myrcene (3.69%), α-pinene (4.08%), Bergamot mint oil (12.73%) [23]	Study—In vitro (antibacterial-) on *Streptococcus faecalis*, *S. aureus*, *Proteus vulgaris*, *Klebsiella pneumoniae*	
				Dose: 10 mg/well	
		Essential oil extracted from seeds [23]		Application: Essential oil	
				[24]	Gum bleeding, Mouth Freshener, Toothache, Toothpowder, Tooth Cleaning [25]
*Ocimum sanctum* Linn. (Holy Basil, Tulsi)	India (Uttar Pradesh) Andaman and Nicobar, Africa, South America, Brazil [26]	Hydrodistillation method, Analyzed-GC-MS	Caryophyllene (22.265%), α-caryophyllene (2.071%), α-pinene (0.125%), copaene (1.637%) and eugenol (15.906%) [27]	In vivo (clinical trial) on humans for efficiency of mouth wash containing tulsi, VAS score for burning sensation—Pre-treatment (5.33 ± 1.80), Post-treatment (2.44 ± 2.10)	Oil extract used to treat toothache
		Essential oil extracted from dried leaves [27]		Dose: 10 mL (thrice)/day–one week),	
				Application: Mouthwash [28]	Dried leaves used to treat gingival and periodontal diseases [29]
*Salvadora persica* L. (Miswak)	India, East, Southern and North Africa, South and West Asia, Arabic Peninsula [30]	Hydrodistillation method, Analyzed-GC-MS	α-caryophellene (13.4%), 1,8-cineole (eucalyptol) (46%), 9-*epi*. -(*E*.)-caryophellene, β-pinene (6.3%) [31]	In vivo (clinical trial) on humans for efficiency of miswak toothpaste against cariogenic bacteria,	Antigingivitis, anti-cariogenic, antiplaque, whitening properties, orthodontic chain preservation and promotion of gingival wound healing [10]
		Essential oil extracted from stem [31]		Dose: twice/day (2 weeks)	
				Application: Toothpaste [32]	
* Eucalyptus globulus * Labill. (Nilgiri)	India: Goa, Gujrat, Haryana, Punjab, Uttar Pradesh. Albina, Spain, Uganda, Cambodia [33]	Analyzed by GC-MS, hydrodistillation extraction method, essential oil extracted from leaves [34]	β-pinene 18.54%, eucalyptol (1,8-cineole) 54.79%, para cymene 1.60%,β-eudesmol 4.68%, α-phellandrene 2.06%, α-pinene 11.46% and gamma-eudesmol 1.20% [34]	*In vivo* (clinical trial) on humans,	For treatment of toothache, sore throat, halitosis in Cameroon mouthwash gargle of *Eucalyptus saligna* is used [3]
				Plaque index score—Baseline (1.485 ± 0.34), After 14 days (1.254 ± 0.58).	
				Dose: 10 mL twice/day (14 days)	
				Application—Gargle, Mouthwash [35]	
*Thymus vulgaris* L. (Thyme, Banajwain)	India (Western Himalayas and Nilgiris), Spain, European countries, Svizzera, France, Italy, Portuguese Republic, Bulgaria, and Ellas [36]	Steam distillation method, Analyzed- GC-MS	Thymol (3.82%), α-thymol (38.71%) camphene (0.13%), caryophyllene (0.915), humulene (0.22%), α-terpineol (0.285) and ρ-cymene (2.77%) [20]	In vitro antimicrobial effect against *Streptococcus mutans* (ATCC 25175),	
		Essential oil extracted from leaves [20]		MIC value (essential oil)—100 μg/mL (1%),	
				Application: Mouthwash [37]	Used in toothpaste, mouth rinse, and aromatherapy for prevention and treatment of oral infection [12,38]
*Azadirachta indica* (Neem)	Afghanistan, Pakistan, India, Sri Lanka, Bangladesh, Myanmar, and China [39]	Hydrodistillation method, Analyzed-GC-MS	Hexadecanoic acid (34.0%), oleic acid (15.7%), 5,6-dihydro-2,4,6-triethyl-(4H)-1,3,5-dithiazine (11.7%), methyl oleate (3.8%), and eudesm-7(11)-en-4-ol (2.7%) [16]	In vivo (clinical trial) on humans,	
				Gingival index score (Chewing stick)—Pre-intervention (0.31 ± 0.44), Post-intervention (0.16 ± 0.29),	
		Essential oil extracted from seeds [16]		Dose—Neem (chewing stick) 20 cm × 20 mm,	
				Application: Chewing	
				[40]	Neem bark extract used in toothpaste or tooth powder. Leaf extract used in mouth rinses [41]
*Acorus calamus* L. (Sweet flag, Vacha)	India, Central Asia, Eastern Europe, Jammu Kashmir, Himachal Pradesh, Manipur, Naga land, Uttarakhand [19]	Steam distillation method, Analyzed- GC-MS			
		Essential oil extracted from leaves [42]	α-Asarone (16.54%), (E)-Methyl isoeugenol (5.06%), γ-Cadinene (3.00)%, α-pinene (2.96%) and Citronellal (2.82%) [42]	In vitro antioxidant activity of *Acorus calamus* (rhizome). DPPH method: IC_50_ value (acetone extract of rhizome)—5 μg/mL [43]	Rhizome part is used for the treatment of dental disorders [44]
*Juglans regia* L. (Walnut, Akhrot)	China, United State, Jammu and Kashmir, Himachal Pradesh, Arunachal Pradesh, Uttarakhand [21]	Hydrodistillation method, Analyzed GC-MS, GC-FIDEssential oil extracted from leaves [45]	Caryophyllene oxide (16.9 to 27.4%),	In vivo (clinical trial) effect of *Juglans regia* dental plaque in humans, Dose: twice/day (3 days),	Bark extract used in oral cavity hygiene, treatment of gingivitis, dental plaque, cleaning of teeth [21]
			β-Caryophyllene (4.0 to 22.5%), Germacrene (1.2 to 9.4%) and	2% ether extract (bark) reported maximum plaque inhibition (32%),	
			β-Pinene (2.8 to 9.5%) [45]	Application: extract directly applied on tooth surface [46]	
*Asparagus racemosus* (Satavari)	Sri Lanka, India, Himalayas, Australia, Africa [17]	Solvent extraction method, Analyzed by GC-MS	Borneol (26.40%), myrtanol (13.72%), pinocarveol (2.37%), 2-ethylhexanol (1.76%) perillaldehyde (8.97%) [47,48]	In vitro antioxidant activity of root extract,	
		Essential oil extracted from aerial parts [47,48]		DPPH method: IC_50_ value (ethanolic extract of root)—468.57 ± 3.002 μg/mL	
				[49]	Antibacterial properties against caries causing oral pathogens [50]
*Melaleuca alternifolia* (Tea tree oil)	India (Ansari et al., 2006), Australia [51]	Steam distillation method, analyzed by GC and GC-MS, essential oil extracted from leaves and terminal branches [52]	Terpinen-4-ol, *p*-cymene, α-terpinene, γ-terpinene, 1,8-cineole, α-pinene and α-terpinol [52]	In vivo (clinical trial) effect of *Melaleuca alternifolia* essential oil on dental plaque in humans in the form of toothpaste along with ethanolic extract of Polish propolis.	
				Comparison after 7 and 28 days of using toothpaste.	
				Result: Approximal plaque index (API)—Before treatment; 64.58 ± 22.38%. After treatment,	
				7 days—(49.00 ± 25.32%, *p* < 0.006) and after 28 days—(39.39 ± 20.60%, *p* < 0.0002) [53]	Periodontitis [54], Relieve from bad breath, bleeding gums, and plaque [55]
*Acacia nilotica* (Babul)	India, Nepal, Pakistan, Arabian Peninsula, Africa, South Africa, Egypt [56]	Hydrodistillation method and analyzed by GC-FID and GC/MS. Essential oil extracted from the bark, leaves [57]	Menthol (34.9%), limonene (15.3%), α-Curcumene (6.9%) and carvacrol (4.1%) [57]	In vitro (antibacterial) on *Lactobacillus acidophilus*, Streptococcus sanguinis, S. salivarius, and Aggregatibacter actinomycetemcomitans.	To cure mouth ulcers [56],
				Dosage: Concentration of extract ranging between 5 and 30 mL in different test tubes. Incubated at 37 °C for 24 h.	To treat toothache and for cleaning teeth [58],
				MIC value of bark extract of *Lactobacillus acidophilus*, Streptococcus sanguinis, S. salivarius, Aggregatibacter actinomycetemcomitans is 40,35,35, and 45, respectively [59]	and for sore throat [60]
*Quercus infectoria* (Baloot)	India, Nepal, Iran, Greece, Syria [61]	Steam distillation (Clevenger apparatus)-Aqueous and ethanolic extract of essential oil from the galls [61]	Tannins 50–70%, gallic acid (2–4%), ellagic acid [61]	In vitro study for dental caries and plaque. Methanol and acetone extracts were screened against bacteria *Streptococcus mutans* ATCC 25175, *Streptococcus salivarius* ATCC 13419, *Porphyromonas gingivalis* ATCC 33277, and *Fusobacterium nucleatum* ATCC 25586.	Used to treat gum infections, gingivitis, and toothache [62]
				MIC value of methanol and acetone extract is 0.16 and 0.63 mg/mL, respectively, while MBC value for methanol and acetone extract is 0.31–1.25 mg/mL and 0.31–2.50 mg/mL, respectively [62]	
*Artemisia dracunculus* (Estragon)	Asia and central Europe	Hydrodistillation method for extraction and GC-MS method for the identification. Extraction of essential oil from aerial parts [61]	Estragol (methyl chavicol), (E)- anethole, capillene, methyl eugenol, (E)-β-ocimene, (E)-α-ocimene, (Z)-β-ocimene limonene, α-pinene, α-terpinolene, isoelemicin, elemicin 5-phenyl-1,3- pentadiyne, α-phellandrene, β-phelland-rene, pulegone, (Z)-artemidin, hinokitiol, and acenaphthene [63]	In vitro(antibacterial) on *Staphylococcus aureus* (ATCC 23235). Concentration—10 µL of tarragon oil tested on agar plate. MIC value is 1250 µg/mL after 24 h of incubation period and MBC value is 2500 µg/mL [63,64]	To treat bleeding gums (gingiva) and bad breath [63]
*Streblus asper* (Koi)	Southern China, India, Sri Lanka, Malaysia, The Philippines, Malaysia, Thailand [65]	Extraction by Hydrodistillation and analyzed by GC-MS method and GC-FID method [66].Essential oil extracted from aerial parts	Leaves: phytol (45.1%), *trans*-farnesyl acetate (5.8%), *α*-farnesene (6.4%), *trans-trans-α*-farnesene (2.0%) and caryophyllene (4.9%) [66]	Leaf extract tested for plaque formation and gingivitis caused by *Streptococcus mutans* and *Actinomycetemcomitans* by using disc diffusion method on agar surface.	Dental caries (Wongkhan et al., 2001), strengthening gums, toothache, and gingivitis [65]
			Stem bark: α-amyrin acetate, β-sitosterol, Strebloside, lupeol acetate, diol, Sioraside, α-amyrin, mansonin, (7’S, 8’S)-trans-streblusol A, (7’S, 8’S)-threo-streblusol B, streblusquinone, 8’R-streblusol C, streblusol E and (8R, 8’R)-streblusol D [65]	The baseline mean of the plaque index is 2.42 in the chlorohexinde group, 1.25 in the placebo group, 2.22 *Streblus asper* alcoholic extract group, and 2.31 in *Streblus asper* aqueous extract group. The baseline mean of the gingival index is 2.12 in the chlorohexidine group, 2.23 in the *Streblus asper* alcoholic extract group, and 2.13 in the *Streblus asper* aqueous extract group was found to be statistically significant *p* ≥ 0.001. Swab from mouth is collected. Duration of test is 21 days [67].	
			Aerial bark: n-Triacontane, β-sitosterol, Stigmasterol, tetraiacontan-3-one, oleanolic acid and botulin [65]		
*Cichorium intybus * (Chicory)	Afghanistan, India, Bulgaria, Italy, Morocco, Iran, Serbia, Jordan, Poland, Serbia [68]	Hydrodistillation method for extraction and analysis is performed by GC-FID method. Essential oil extracted from aerial part [69]	Carvacrol (50.1%), cinnamic aldehyde (12.4%), thymol (13.3%), camphor (4.4%), linalool (3.9%), carvone (4.1%) and terpineol (2.1%) [69]	In vitro agar diffusion method (antibacterial) on *Staphylococcus aureus*, *Bacillus subtills*, *Escherichia coli, and Salmonella typhi* causing plaque formation, tooth caries, and gingivitis. Methanolic extract of leaf and root show maximum inhibition at 200 mg/mL concentration [70]	To break up molars (with cavities), plaque, gingivitis, and tooth decay [71]
*Vitex negundo* (Nirgundi)	Afghanistan, India, Sri Lanka, Pakistan, Thailand, eastern Africa, Malaysia, Madagascar [72]	Hydrodistillation method for extraction and analysis by GC and GC-MS method. Essential oil extracted from leaves, flowers and dried fruits [73]	δ-guaiene, epoxide, ethyl-hexadecenoate, guaia-3,7-dienecaryophyllene epoxide, α-selinene, caryophyllene epoxide, germacren-4-ol β-selinene, (E)-nerolidol, α-cedrene, germacrene D, hexadecanoic acid, p-cymene, valencene, germacrene, D viridiflorol (19.55%), β-caryophyllene (16.59%), sabinene (12.07%), γ-terpinene (2.21%), 4-terpineol (9.65%), caryophyllene oxide (1.75%), 1-oceten-3-ol (1.59%), 1-oceten-3-ol (1.59%) and globulol (1.05%) [74]	In vitro study on *Streptococcus mutans*, *Streptococcus sanguis,* and *Staphylococcus aureus.* The aqueous, methanolic, and petroleum ether extract of *Vitex negundo* were tested for their antibacterial activity using well diffusion method. Concentration: 200 mg/mL. Maximum inhibition zone is shown by methanolic extract is 23 mm [75]	Toothache, throat pain, mouth ulcers (Ullah et al., 2012). The decoction prepared from the leaves of *Vitex negundo* is used for gargling in the treatment of mouth ulcers [76]
*Rosmarinus officinalis *(Rosemary)	South Europe, India, Mediterranean basin [77]	Hydrodistillation for extraction (Elyemni et al., 2019) and analysis by HPLC and gas chromatography. Extraction of essential oil from leaves [78]	Borneol (1.5–5.0%), camphor (5–31%), pinene (9–26%), 1,8-cineol (15–55%), camphene (2.5–12.0%), pinene (2.0–9.0%), limonene (1.5–5.0%), myrcene (0.9–4.5%), verbenone (2.2–11.1%) and caryophyllene (1.8–5.1%) [79]	Clinical trial on the action of toothpaste made from the extract of *Rosmarinus officinalis* on humans divided into two groups (experimental and controlled), assessed at baseline and 30 days after the study using the gingival bleeding index (GBI) and the plaque index (PI).	Plaque [79], dental caries [80]
				Results: reduction of 38% in the risk of gingival bleeding (relative and absolute)	
				And reductions in bacterial plaque is 22.7% [80]	
*Embelia ribes* (Vaibidang)	Sri Lanka, China, India, Malaysia [81]	Soxhlet extraction and analysis by FT-IR, DSC, UV-visible, NMR, X-ray diffraction, and TGA method. Extraction of essential oil from berries [82]	Embelin, embolic acid, rapanone [81] and vilangin [83]	The extract of *Embelia ribes* at a concentration of 500 mg/50 mL reported 12 mm diameter of zone of inhibition against test organism *Bacillus subtilis,* causing periodontitis and tooth decay [84].	Dental cavities, as mouthwash, gum infection, and tooth decay [85]
*Spilanthes* species	Tropical Africa, South America, Tropical America, North Australia, Africa, Malaya, Borneo, India, Sri Lanka [86]	Simultaneous distillation extraction method for isolation and GC-MS method for analysis. Essential oil extracted from stem, leaves, and flowers [86]	α-and β-bisabolenes, α-caryophyllene, β-caryophyllene, cadinene, *N-*(isobutyl)-2E,6Z,8E-decatrienamide, *N-*(isobutyl)-6Z,8E-decadienamide, *N-*(2-methylbutyl)-2E,6Z,8E-decatrienamide, decatrienamide, *N-*(2-phenylethyl)-2E,6Z,8E-decatrienamide [86]	Chewing on the flower heads and roots has shown to decrease gum inflammation and have been used in the treatment of periodontitis [87]	Toothache, throat complaints [88,89], Tooth decay [90], Sore throat, gum infection [91], Gingivitis [92]
*Nigella sativa* L. (Kalonji)	The Middle East, southern European continent, North Africa, India: Bengal, Bihar, Gangetic plains, Himachal Pradesh, Assam, Maharashtra, and Punjab [93]	Essential oil from seeds extracted using solvent extraction and SFE method and analyzed by GC-MS [94]	Carvacrol (5.8–11.6%), longifolene (1.0–8.0%), ρ-cymene (7.1–15.5%), t-anethole (0.25–2.3%), 4-terpineol (2–6.6%) and thymoquinone (27.8–57.0%) [94]	Clinical trial on efficacy of 0.2% thymoquinone oral gel (topical) in treatment of periodontitis: heathy female and male patients with at least 2 periodontally involved sites (≥5 mm), n = 20.	
				Dose: repeated from baseline up to 4 weeks.	
				Result showed reduction in GI, PI and PPD levels [95]	Essential oil having anticarcinogenic, antioxidant and antimicrobial properties [96]

**Table 3:** Clinical trials on the effect of antioxidant extracts from medicinal plants in oral health.

Title	Extract and Dose Used	Objective	Location	Main Finding of the Study	Reference
Comparative evaluation of efficacy of 4% tulsi extract fluoridated and placebo dentifrices against gingivitis and plaque: a triple-blind RCT	4% ethanolic extract (tulsi dry leaves), Dose: twice/day (21 days), Application:Toothpaste	To assess and compare the antigingivitis and antiplaque effect of fluoridated, placebo dentifrice (PD) and 4% tulsi leaf extract dentifrice among 14–15-year-old school children.	Davangere city, India	Maximum reduction in dental plaque (*p* = 0.01) and gingivitis score (*p* = 0.001) in 4% tulsi dentifrice compared to PD.	[156]
Comparative clinical effects of *Salvadora Persica* oral rinse and phenolic commercial mouth wash on human oral health: a triple-blind RCT	*Salvadora persica* oral rinse 50% conc., Dose: 15 mL twice/day (6 months), Application: gargle, mouthwash	To compare the clinical effects of *Salvadora persica* oral rinse and commercial phenolic mouth wash on oral health status of socially deprived madrasa girls 18–22 years old.	Multan city, Pakistan	With no statistical difference in gignival and plaque scores, *Salvadora persica* oral rinse is equally effective as phenolic mouth wash.	[157]
Antiplaque effect of hiora-GA gel, spirogyl gum paint, and tooth and gums tonic in comparison with chlorhexidine M gel: a double-blind RCT	*Eucalyptus globulus* extract (tooth and gums tonic), Dose: twice/day (90 days), Application: gel directly applied on tooth surface	To compare the efficacy of three different herbal products in gingival inflammation, bacterial count, and reducing plaque in comparison with chlorhexidine M gel among participants with moderate to severe periodontitis.	Osmania Dental College and Hospital, Hyderabad, India	The mean gignival and plaque scores were decreased at different intervals, and no significant difference is oserved in efficacy of gel compared to chlorhexidine.	[158]
Effect of 2.5% sodium hypochlorite versus neem as root canal irrigants on the intensity of post-operative pain and the amount of endotoxins in mandibular molars with necrotic pulps: RCT	Neem (root canal irrigant), Dose: one time each followed by two root canal treatments	To assess the efficacy of 2.5% NaOCl versus neem as root canal irrigants on amount of endotoxins and intensity of post-operative pain following root canal treatment of mandibular molars with necrotic pulps	Cairo University, Egypt	In neem group, mean pain scores were lower as compared to 2.5% NaOCl, and neem group reduced endotoxin level by 18% in comparison with pre-instrumentation samples.	[159]
Clinical effect of *Juglans regia* on the developing dental plaque: RCT	2% ether extract (bark),	To assess the clinical effect of 2% aqueous extract, 2% and 3% concentration of ether fractions in propylene glycol and petrol-ether extract of bark of *Juglans regia* against developing plaque	Faculty of Dental Sciences, C. S. M. Medical University, Lucknow, India	2% ether extract of *Juglans regia* showed maximum antiplaque activity of 32.12% (*p* < 0.001).	[46]
	Dose: twice/day (3 days),				
	Application: extract directly applied on tooth surface				
Intrapocket application of *Melaleuca alternifolia* tea tree oil (TTO) gel in the treatment of stage II periodontitis: a phase 2 clinical trial	5% TTO gel and SRP, Dose: 0.5 mL gel, Application: gel directly applied on dental pockets	To assess biochemically and clinical the effect of intrapocket application of TTO gel and scaling and root planing (SRP) in the treatment of stage II periodontitis and to correlate biochemical levels with clinical response	Faculty of Dentistry, Alexandria University	TTO gel adjunctive to SRP is found to be effective in treatment of stage II periodontitis.	[160]
			Alexandria, Egypt		
Effect of a Toothpaste/Mouthwash Containing Carica papaya Leaf Extract on Interdental Gingival Bleeding: A Randomized Controlled Trial	Carica papaya leaf extract	To study the comapartive effectiveness of dentifrice having papaya leaf extract to a commercially available sodium lauryl sulfate-free enzyme-containing dentifrice in management of gingival bleeding	Dental Faculty, University of Granada, Spain	Papaya leaf extract dentifrice/mouthwash provides an efficacious and natural alternative to sodium lauryl sulfate-free dentifrice and reduces gingival bleeding.	[161]
	Application: Mouthwash and tooth paste				
Use of an antiviral mouthwash as a barrier measure in the severe acute respiratory syndrome coronavirus 2 (SARS-CoV-2) transmission in adults with asymptomatic to mild COVID-19: a multicenter, randomized, double-blind controlled trial	ß-cyclodextrin and citrox (bioflavonoids) (CDCM)	To determine if commercially available mouthwash with CDCM could decrease the SARS-CoV-2 load from saliva	Hospital Centers, France	CDCM had a significant beneficial effect on reducing SARS-CoV-2 salivary viral load in 280 adults with asymptomatic or mild COVID-19, 4 h after the initial dose.	[162]
	Application: Mouthwash				

Randomized clinical trial (RCT), placebo dentifrice (PD), tea tree oil (TTO).
